# Reply to Smith and Siegel: Most lithium hops in paddlewheel-claimed conductors occur without spatially and temporally correlated anion-group rotations

**DOI:** 10.1073/pnas.2423194122

**Published:** 2025-02-24

**Authors:** KyuJung Jun, Gerbrand Ceder

**Affiliations:** ^a^Department of Materials Science and Engineering, University of California, Berkeley, CA 94720; ^b^Materials Sciences Division, Lawrence Berkeley National Laboratory, Berkeley, CA 94720

We appreciate the engagement from Smith and Siegel (1) on the topic of the “paddlewheel effect” and are pleased to see alignment regarding the absence of a paddlewheel effect, in which large-angle anion-group rotations directly propel lithium hops. In our paper (2), we clearly state the ambiguity surrounding the term “paddlewheel” to describe lithium transport. We believe that science is not well served by this vague term or its regular redefinition which is why we distinguish three types of anion-group rotation events: large-angle rotations (n-fold rotation returning to a rotationally invariant configuration), librations (e.g., rotational vibrations), and static changes of orientation in response to the change of Li occupancy. We directly evaluate the spatial and temporal correlation between such polyanion rotations and Li hops.

Our results appear undisputed by Smith and Siegel: the correlation between anion dynamics and lithium hopping is minimal and does not account for the majority of Li hopping. We find that only static changes in polyanion orientations are correlated to the Li hopping events through a relaxation of the sulfur near the lithium hopping pathway. Such anion relaxations are present in virtually all conductors, and no special physics or new mechanism needs to be invoked to describe it. Hence, we object to the term paddlewheel effect as it neither clarifies nor distinguishes itself from the relaxation that accompanies Li diffusion in other materials.

We performed rigorous event-based detection and statistical analysis and demonstrated that most lithium hops do not exhibit spatial or temporal correlation with significant anion-group rotation (orange lines, Fig. 5 *C* and *D* and *SI Appendix*, Figs. S20–S45). Coupled rotations/hopping, while observable, occur infrequently and represent only a small fraction of total transport events. Without statistical significance of such coupled motion in enhancing Li diffusion, we believe that referring to its “existence” as a paddlewheel effect is not justified.

With regard to the concerns about our choice of density for amorphous Li_3_PS_4_ we reiterate that we used experimentally measured densities for fully densified amorphous samples that show no evidence of crystallization [Sakamoto (3) gives 1.88 g/cm^3^ and Tatsumisago (4) reports 1.85 g/cm^3^]. Given the absence of a “unique” amorphous structure, we believe this is the most prudent approach. Hence, we used 1.8 g/cm^3^ in the main text and included an upper bound model of 2.0 g/cm^3^. Smith and Siegel cite an earlier work (5) reporting crystallization in high-density amorphous samples, but a follow-up study by the same authors, including Smith and Siegel (3) demonstrates that modifying densification conditions can yield high-density amorphous materials without crystallization, as explicitly stated.

In conclusion, we reaffirm that Li hopping shows minimal correlation with the dynamics of polyanion rotation, and there is nothing special about Li migration in (isolated) polyanion-based solids. As in any conductor, Li hopping is accompanied by structural relaxation. In our studies as well as in those by Smith et al. this local relaxation takes the form of small polyanion reorientations. Hence, there is no need to invoke the already-ambiguous terminology of the paddlewheel mechanisms.
